# Peer review: the imprimatur of scientific publication

**DOI:** 10.1113/EP092108

**Published:** 2024-08-14

**Authors:** Ronan M. G. Berg, Karyn L. Hamilton, Joanne Fiona Murray, Peying Fong

**Affiliations:** ^1^ Centre for Physical Activity Research Copenhagen University Hospital – Rigshospitalet Copenhagen Denmark; ^2^ Department of Clinical Physiology and Nuclear Medicine Copenhagen University Hospital – Rigshospitalet Copenhagen Denmark; ^3^ Department of Biomedical Sciences, Faculty of Health and Medical Sciences University of Copenhagen Copenhagen Denmark; ^4^ Neurovascular Research Laboratory, Faculty of Life Sciences and Education University of South Wales Pontypridd UK; ^5^ Department of Health and Exercise Science, Center for Healthy Aging Colorado State University Fort Collins Colorado USA; ^6^ Centre for Discovery Brain Sciences, Edinburgh Medical School, Biomedical Sciences University of Edinburgh Edinburgh UK; ^7^ Department of Anatomy and Physiology Kansas State University College of Veterinary Medicine Manhattan Kansas USA

## 1 INTRODUCTION

At *The Journal of Physiology* and *Experimental Physiology*, we continually rely on the keen support from expert referees to provide external peer review of submitted manuscripts (Forsythe, 2017). This is a mandatory step in the publication process, as it is in all other established journals within the biomedical and allied sciences. Indeed, even with the digitalization of scientific communication over the past three decades, the peer‐reviewed scientific article remains the primary outlet for disseminating research (Nicholas et al., 2015). As physicist John Ziman noted in his widely cited book, *Public Knowledge*, ‘the referee is the lynchpin about which the whole business of Science is pivoted’ (Ziman, 1968).

Recent significant changes in the publication landscape have posed challenges to conventional academic publishing models, challenges of which we, as editors of *The Journal of Physiology* and *Experimental Physiology*, are acutely aware. These changes include the open access model of publishing and the use of preprint servers. Over the last two decades there has been a move from the traditional publishing model to the open access publishing model; the major difference between the two models being that the reader has been replaced by the author as the primary source of income for the publisher. Both the scientific community and the public benefit from this more equitable approach to disseminating science. The use of preprint servers allows researchers to upload manuscripts to disseminate findings before undergoing formalized peer review, in an effort to accelerate the accessibility of research and its wider availability to the public. Both these changes align with policies from *UK Research and Innovation*, the *European Commission* and the *US White House Office of Science and Technology Policy*, which all encourage making publicly funded research results freely and immediately available to both the wider scientific community and the public.

In light of these developments, some suggest that the peer review process, and perhaps even the classical scientific journals themselves, will become obsolete (DeMaria, 2023; Lu et al., 2024). Starting in 2025, the Bill & Melinda Gates Foundation, one of the major funding sources of biomedical research programmes, will require its grant holders to make their research publicly available as preprints while, at the same time, all financial support to pay for open access fees to peer‐reviewed journals will cease (Lenharo, 2024); these two actions by this influential Foundation will expectedly further advance the move away from the peer review process and classical scientific journals. We suspect that misuse of the open access process as a business model by some publishing companies, which creates an economic incentive to accept more or less anything for publication in so‐called ‘predatory’ journals (Chandra & Dasgupta, 2024), is probably the reason behind the Foundation's decisions. Those publishing companies that misuse the open access process render peer review a mere formality without any substantive value while, by charging authors for publication, generate revenue for their stakeholders through the misappropriation of funds intended for biomedical sciences.

At *The Journal of Physiology* and *Experimental Physiology*, we welcome these changes to make research more accessible to the wider scientific community and the public. However, like many other official journals of scientific societies and established journals, we are concerned that the importance of the peer review process is being devalued. Here, we make the case that, in light of these developments, pre‐publication peer review of scientific research, with the scientific journal as the outlet, is more important than ever, and furthermore, that it is our collective duty as scientists to contribute to this process.

## 2 ORIGINS AND PRACTICE OF EXTERNAL PEER REVIEW

The historical origins of peer review are often traced back to Henry Oldenburg (1618–1677), the secretary of the Royal Society in the late 17th century, who is credited with introducing external refereeing to the *Philosophical Transactions* (Csiszar, 2018). However, it was not until 1752, when the Royal Society created the Committee of Papers to vet submissions, that formal external reviews were obtained, mostly to ensure that the responsibility for the veracity of the content did not reside with the Royal Society itself. In the 19th century, external reviews from renowned scientists began to be published along with the original contributions in *Philosophical Transactions*, mostly as a publicity stunt to enhance the visibility of the published scientific discoveries.

The practice of referring articles to external referees continued through the 19th and early 20th century (Csiszar, 2018). However, the standardization of the peer review process emerged from the significant increase in research funding following World War II. With this came the need for expert authority and objectivity across rapidly expanding and increasingly diverse scientific disciplines, while a commercially successful and internationally oriented journal‐publishing industry emerged (Csiszar, 2018). Concurrently, there was a concerted effort to establish standard practices for journal editors, addressing the implications of commercial firms' growing influence on journal publishing and their relationships with learned institutions and individual researchers (Fyfe et al., 2017). The use of external peer review thus became a means for maintaining the integrity of scientific discourse amid an influx of scientific claims, often entangled with commercial interests.

The exact time when external peer review was first introduced at *The Journal of Physiology* and *Experimental Physiology* remains unclear. It is improbable that John Newport Langley, arguably the most notorious Editor‐in‐Chief in the history of *The Journal of Physiology*, saw the need for input from external parties. During his tenure from 1894 to 1925, he largely managed the journal single‐handedly, providing authors with extensive feedback and often personally rewriting sections of manuscripts before their acceptance (Bailey et al., 2023). At *Experimental Physiology*—originally named the *Quarterly Journal of Experimental Physiology*—the founding Editor‐in‐Chief, Edward Sharpey‐Schafer, who served from 1908 to 1933, implemented a starkly different editorial policy. In direct contrast to Langley's method, which Langley would have certainly deemed very laissez‐faire, Sharpey‐Schafer aimed for published articles to reflect the ‘author's own voice’, thus minimizing any editorial intervention (Bailey et al., 2023). Inadvertently, this approach also made input from external parties unnecessary.

However, sometime after the tenure of Langley, and in line with the aforementioned developments, by 1944 *The Journal of Physiology* had formulated its first official ‘instructions for referees’. The *Quarterly Journal of Experimental Physiology* later implemented this practice as an editorial strategy to align with the editorial policies of *The Journal of Physiology*, even before it was taken over by The Physiological Society in 1981. Nevertheless, for the following decades, manuscript review mainly continued to be internal (i.e. by the editorial board members), with only rare consultation of external referees. In 1967, the instructions were updated, and efforts made to make wider use of external referees to ‘improve the quality of the published work’. When external peer review became mandatory in *Nature* in 1973, it marked the beginning of a widespread requirement for external peer review in all established scientific journals (Baldwin, 2015). Admittedly, *The Journal of Physiology* and *Experimental Physiology* were slow to adopt this practice, as it was not until the 1990s that they made external peer review mandatory for all submitted manuscripts.

Although external peer review is now mandatory within all established journals in our field, it is also widely criticised for being slow and costly. Additionally, it is often coined as inherently conservative and censorial, without necessarily detecting major errors, plagiarism and fraudulent research (DeMaria, 2023; Raff et al., 2008; Siegel, 2008). Indeed, an author's experience of peer review can sometimes resemble a Kafkaesque trial, where the author is subjected to the seemingly arbitrary whims of a faceless judge with the power to make or break their scientific contributions. Consider, for instance, the persistent—albeit unfounded (Worsham et al., 2022)—rumours of the particularly fierce and unpredictable ‘Reviewer 2’, who may resort to *ad hominem* attacks (Watling et al., 2021). Perhaps this more than anything reflects that the publication process, including peer review and the exact role of the referee, is somewhat opaque. We note that *The Journal of Physiology* has recently begun to publish peer review histories of its publications online. While this serves as an incentive for referees to provide exemplary review reports, a previous study indicates that this practice does not in itself affect the quality of the review reports (van Rooyen et al., 2010), even as it intends to make the review process more transparent to readers.

To clarify the roles within the peer review process, it is essential to understand that external referees are *not* the decision‐makers regarding the ultimate fate of a manuscript (Glonti et al., 2019). The task rests solely with the editor. In this context, the editor acts as the judge, while the referee fulfils the roles of both the devil's advocate and the jury. As the devil's advocate, the referee identifies weaknesses and limitations in the study design and any issues in the interpretation of data, their implications and generalizability as presented in the manuscript, of which the author(s) may or may not already be aware. Objectivity is a key aspect when it comes to the communication of science, including the presentation of methods, results and interpretation. However, although we all strive to be as objective as possible, any scientist is potentially biased when presenting and interpreting their own ideas and findings, including their implications and importance. As the jury, the referee advises the editor through an assessment of the quality of the research and the manuscript itself. While grounded in area‐specific expertise, there is still an element of inherent subjectivity—or more precisely, an ‘unobjectifiability’—in these judgments that likely forms the primary source of the criticism faced by the peer review process. In contrast, other aspects, such as feedback to improve the transparency and clarity of reporting, can be assessed more objectively.

We have not (yet) conducted studies to assess the impact of peer review on study quality in The Physiological Society's journals, although the paper referral trajectory from *The Journal of Physiology* to *Experimental Physiology* and *Physiological Reports* would offer unique insights. However, studies in other journals have reported that peer review substantially improves the quality of scientific manuscripts, particularly when it encompasses subjective (i.e. unobjectifiable) and complex assessments of specific aspects of the manuscript, such as the generalizability of the results and study limitations (Goodman et al., 1994; Pierie et al., 1996), as well as more objectively clear aspects, such as statistical reporting (Carneiro et al., 2020; Gardner & Bond, 1990). Nevertheless, no consensus exists on how to measure the effects of peer review, particularly concerning the unobjectifiable aspects of the referee's quality assessment (Jefferson et al., 2002; Kassirer & Campion, 1994). Moreover, since most studies on peer review have been carried out in small numbers of specialist journals—with the risk of potential selection bias from the peer review of rejected papers that are ultimately submitted elsewhere—there is currently little empirical evidence supporting the effectiveness of peer review in ensuring article quality (Jefferson et al., 2007).

## 3 THE PREPRINT REVOLUTION

By definition, a preprint is a complete manuscript posted to a preprint server by authors before it undergoes peer review and is published in a scientific journal. Its use has been common in communities such as physics and mathematics for over 30 years. This enables authors to receive timely feedback and comments on their research before submission to a peer‐reviewed journal, while also facilitating the rapid dissemination of their research, and enabling them to claim the provenance of an idea. Recently, spurred by the creation of new repositories through scientist‐driven initiatives, scientists within the biomedical sciences have become more adept at using preprints (Flanagin et al., 2020). In 2020, the urgent threat of a global pandemic catapulted the use of preprint servers as a means to quickly disseminate scientific findings into the public sphere (Fraser et al., 2021).

While the rapid dissemination of scientific findings through preprint servers was clearly a necessity during the COVID‐19 pandemic (Flanagin et al., 2020), significant caveats of this publishing model were also uncovered during this period. First, non‐scientists often perceived preprints to be as credible as peer‐reviewed articles (Wingen et al., 2022), and widespread media coverage and social media discussions of non‐peer‐reviewed research susceptible to misinterpretation or exaggeration thus ensued (Brierley, 2021; Fleerackers et al., 2022, 2024). While this may to some extent imply a temporary shift in journalistic norms (Fleerackers et al., 2024), the increased coverage of preprints seen during the pandemic probably contributed to the spread of fake news and conspiracy theories (Fraser et al., 2021). Early in the pandemic, advocacy by politicians and physicians for specific treatments based on data provided only in preprints may furthermore have complicated the conduct of subsequent randomized clinical trials (Flanagin et al., 2020).

Another important aspect to consider is the actual quality of the studies posted as preprints during the pandemic. Although research quality is difficult to quantify as mentioned above, the fact that a substantial proportion of preprints are never published in peer‐reviewed scientific journals suggests that many may represent studies of lower quality, unable to endure formal peer review (Añazco et al., 2021). It is also worth noting that online feedback from the scientific community, one of the potential benefits of the preprint publishing model intended to provide pre‐submission peer review, is limited for preprints posted both during and after the pandemic, and furthermore, such commentary is rarely incorporated into the articles when submitted (Brierley, 2021; Carneiro et al., 2023; Kodvanj et al., 2022). However, there are some studies that indicate that clinical studies posted as preprints and later published in peer‐reviewed journals showed consistent study characteristics, results and interpretations (Janda et al., 2022). In these instances, the findings suggest that many preprints report results that align with those in the final peer‐reviewed publications. The main differences reported between the preprint and the final publication in a scientific journal often relate to improvements in transparency and clarity in the presentation of methods and results (Carneiro et al., 2020), which thus seem to be specifically enhanced by the peer review process.

## 4 CONCLUDING REMARKS

The peer review process holds a patchy reputation, and its benefits in terms of improving the quality of published research are challenging to document thoroughly. However, the fundamental argument in favour of peer review is not empirical but normative, underpinning the very integrity of biomedical research. The publication of research fundamentally aims to share new knowledge. John Ziman argued that the goal of all scientific research is to contribute to the consensus of universally accepted knowledge (Ziman, 1968), both with the scientific community and the wider society, but in doing so, it carries noteworthy stakes: it facilitates communication, legitimizes findings, advances careers, influences funding priorities and guides future research. To justify this approach, the trustworthiness of the scientific outlet is crucial. That is: *What is knowledge without trust?*

In an international project sponsored by the Alfred P. Sloan Foundation, ∼4000 academic researchers across disciplines identified peer review as the singular most important factor that determines the trustworthiness of scientific information in the complex, information‐rich digital environment (Nicholas et al., 2015). John Ziman went even further to state that peer review not only confers trustworthiness on scientific articles but also imbues them with an ‘imprimatur of scientific authenticity’ (Ziman, 1968). Thus, peer review has transformed from originally being a means of publicity in the 18th and 19th centuries to a mark of credibility indicating independence from commercial and other external interests in the first half of the 20th century, something that remains equally relevant today. From the 1970s onwards, it has furthermore served as a tag of inherent trustworthiness, thereby distinguishing scientific from non‐scientific publications. This does not mean that peer review necessarily prevents fraudulent or fake science from being published, nor does its failure to do so disqualify it. Nor does it mean that peer review should be censorial in preventing the publication of ideas that turn out to be incorrect or studies that reach the wrong conclusions. Publication of peer‐reviewed findings and conclusions are exposed to open and transparent review by the scientific community (Tipton, 2019). Some would argue that only by objectively investigating and openly debating ideas can we truly test them and, thereby, either elevate or dismantle them. But what does peer review add to this process?

When we serve as referees, we make an altruistic contribution to the advancement and sharing of knowledge. As representatives of the scholarly community, we must recognize that peer review is not merely a task but a distinct discipline within scientific research. This discipline should be approached professionally, embodying mentorship rather than mere judgment. The aim is to transcend the mythical ‘Reviewer 2’ by providing unbiased, expert advice and ensuring manuscripts are enhanced from submission to publication. Ideally, constructive peer review fosters a productive dialogue between author and referee, setting actionable goals that refine the final product. In optimal scenarios, this process can uncover scientific nuances that profoundly transform the final work. When published in a journal, the peer review serves as an imprimatur, lending the authority of the scientific community—as represented by the external and ideally unbiased expert referees as well as by the journal editorial board—to authenticate the paper as *science*. This indicates that the work qualifies to serve for further scientific discussion and replication, as part of the ‘self‐correcting nature of science’ (Whipp, 2010).

Recapitulating the words of former Editor‐in‐Chief of *Experimental Physiology*, Mike Tipton, we are not convinced that the publication or presentation of research that has not been peer‐reviewed is beneficial for science, students of science, or the pursuit and promulgation of truth (Tipton, 2019). Given that preprints are posted prior to any peer review, they cannot and should not be considered a definitive means of scientific communication. While preprints offer the clear benefit of rapid access to new findings that can stimulate topical discussions among scholars, they should *complement* the publication of research in peer‐reviewed scientific journals. Importantly, they are neither essential to nor can they replace peer‐reviewed research publications. Thus, while the scientific community may understand the nuances and biases of non‐peer‐reviewed literature, the general public may not, as exemplified by the collective societal experiences ensuing from the surge of preprints and their misuse during the COVID‐19 pandemic. This is not to suggest that we oppose the public availability of science; however, it has not been authenticated as science until it has undergone peer review. Rather than using preprint servers for this purpose, this is more credibly achieved through responsible open access initiatives, as recently implemented in *Experimental Physiology* (Bailey & Stewart, 2022). In our opinion, dissemination to the public and policy changes should not be pursued until authentication by peer review has occurred, a demarcation that is crucial in maintaining the trustworthiness of scientific communication.

## AUTHOR CONTRIBUTIONS

Peying Fong: conception and idea, first draft, revisions. Karyn L. Hamilton: conception and idea, first draft, revisions. Ronan M. G. Berg: first draft, revisions. Joanne Fiona Murray: revisions. All authors have read and approved the final version of this manuscript and agree to be accountable for all aspects of the work in ensuring that questions related to the accuracy or integrity of any part of the work are appropriately investigated and resolved. All persons designated as authors qualify for authorship, and all those who qualify for authorship are listed.

## CONFLICT OF INTEREST

The authors have no conflict of interest to declare.

## FUNDING INFORMATION

The Centre for Physical Activity Research (CFAS) is supported by TrygFonden (grants ID 101390 and ID 20045). The funders had no role in study design, data collection and analysis, decision to publish, or preparation of the manuscript.
